# A Textbook on Diseases of the Ear, Nose and Throat

**Published:** 1902-05

**Authors:** 


A Textbook on Diseases of the Ear, Nose and Throat. By Charles H. Burnett, M D., E. Fletcher Ingals, M.D., and James E. Newcomb, M.D. Octavo, pp. 734. Illustrated. Philadelphia and London: J. B. Lippincott Co. 1901. [Price, $5.00.]

This work is divided into three parts, each of which has been written by an experienced and well-known teacher and writer on these subjects. The first part, diseases of the ear, is by Dr. Charles H. Burnett, of Philadelphia, one of the most celebrated and most skilful aurists in the United States. The second part, diseases of the nose and naso-pharynx, is by Dr. E. Fletcher Ingals, assisted by Dr. O. T. Freer, both of Chicago. Dr. Ingals has been known for many years as one of the best teachers and writers of his specialty. The third part, diseases of the pharynx and larynx, is by Dr. James E. Newcomb, who is recognised as one of the most capable teachers and writers in his specialty in New York City.

The thoroughness of this book is evident on every page that one reads. Nothing that pertains to disease of the ear, nose or throat seems to have escaped the consideration due it by the authors. One is surprised that so much valuable information could have been brought together in so clear a manner, in so small a book; and while it may be valuable as a text-book for students and general practitioners, it is equally valuable to the specialist as a work of reference.

The writer of the chapters devoted to diseases of the ear had, perhaps, the best opportunity of describing new methods of treatment and other advances, for his treatments "are," as he says in the preface to the book, "at once the newest and accepted as the best by the leading specialists." Otology has undergone more changes than has either rhinology or laryngology during the last decade. Every page of this portion of the book has upon it the stamp of the progress which otology is making.

The chapters devoted to anatomy and physiology of the ear are well worth reading; while the latter portion of this part of the book, descriptive of purulent diseases of the middle-ear and their complications, is almost exhaustive.

The last two divisions of this work are equally concise and exact, but the authors have not had the opportunity to bring new material into the text which Dr. Burnett had; for rhinology and laryngology have not been making the rapid strides forward that otology has. Yet every chapter might be dwelt upon, for good points are evident everywhere; for example, the treatment of deflection of the nasal septum, in the chapter on diseases of the nasal septum, is well worth reading by all who have not strong prejudices in favor of a particular operation on the septum. We do not believe that a more reasonable presentation of the comparative merits of the various operations upon the septum has ever been written. Equally striking is Dr. Newcomb's description of the various methods of treating tuberculosis of the larynx.

These are only illustrations, other examples of equal merit might be multiplied, but it is not necessary to do so. The merits of this book will manifest themselves at once to the reader.—W.S.R.

